# Fundus autofluorescence abnormalities can predict fluorescein angiography abnormalities in patients with chronic central serous chorioretinopathy

**DOI:** 10.1007/s00417-023-06042-z

**Published:** 2023-04-10

**Authors:** Danial Mohabati, Camiel J. F. Boon, Carel B. Hoyng, Konstantine Purtskhvanidze, Johann Roider, Elon H. C. van Dijk

**Affiliations:** 1 Department of Ophthalmology, Leiden University Medical Center, Albinusdreef 2, 2333 ZA Leiden, the Netherlands; 2 Department of Ophthalmology, Amsterdam University Medical Centers, Amsterdam, the Netherlands; 3 Department of Ophthalmology, Donders Institute for Brain, Cognition, and Behaviour, Radboud University Medical Center, Nijmegen, the Netherlands; 4 Department of Ophthalmology, University Medical Center, University of Kiel, Kiel, Germany

**Keywords:** Retina, Imaging, Macula, Chronic central serous chorioretinopathy, Fluorescein angiography, Fundus autofluorescence

## Abstract

**Purpose:**

This study is to assess the possible correlation between findings on fundus autofluorescence (FAF) and fluorescein angiography (FA) in patients with chronic central serous chorioretinopathy (cCSC).

**Methods:**

This multicentre retrospective cohort study included 71 cCSC patients (92 eyes) with at least 6 months of follow-up, who had a FAF-FA imaging discrepancy larger than 0.5 optic disc diameters in size in the corresponding areas of hyperfluorescent abnormalities. A comparison was performed between progression in size of areas of hyperautofluorescent retinal pigment epithelium (RPE) abnormalities on FAF (HF-FAF) and the hyperfluorescent areas on FA (HF-FA) at first visit and last visit. The possible correlations were estimated between FAF-FA discrepancy and disease characteristics.

**Results:**

The median area of HF-FAF at first visit was 7.48 mm^2^ (1.41–27.9). The median area of HF-FA at first visit and last visit was 2.40 mm^2^ (0.02–17.27) and 5.22 mm^2^ (0.53–25.62), respectively. FAF-FA discrepancy was associated with follow-up duration and the area of HF-FAF at first visit. A mathematical algorithm for grading FAF-FA discrepancy in time was suggested, which predicted the enlargement of hyperfluorescent RPE abnormalities on FA in 82.6% of cases.

**Conclusion:**

There is a statistically significant relationship between the areas of HF-FAF and HF-FA in cCSC patients with FAF-FA imaging discrepancy at first presentation. Long-term changes in RPE alterations in cCSC on FA can be predicted based on baseline HF-FAF and follow-up duration.


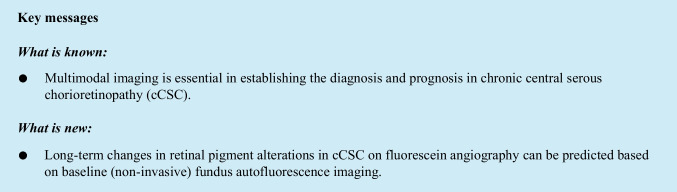


## Introduction

Central serous chorioretinopathy (CSC) is a chorioretinal disease, characterized by a serous retinal detachment due to accumulation of subretinal fluid (SRF), typically affecting middle-aged male patients [1]. In this disease, choroidal thickening and hyperpermeability have been hypothesized to lead to dysfunction of the retinal pigment epithelium (RPE) and serous fluid leakage into the subretinal space [2].

In most cases of acute CSC, SRF resolves spontaneously within 4 to 6 months, often without residual visual impairment [3]. Chronic CSC (cCSC), characterized by persistent or recurrent accumulation of SRF, may lead to severe and permanent visual symptoms and to decreased quality of life [4, 5]. Despite the fact that indocyanine green angiography (ICGA) reveals important information on CSC pathogenesis by visualizing the underlying widespread choroidal abnormalities, fluorescein angiography (FA) is still the most widely used technique to obtain information on the area and morphology of RPE abnormalities and the leakage through the RPE in CSC [1].

Several studies described using short-wave fundus autofluorescence (FAF) for the evaluation of retinal abnormalities in CSC patients and compared them with other imaging modalities [6–13]. Next to optical coherence tomography (OCT)—providing accurate information on the accumulation of SRF and intraretinal fluid—FAF was also found to establish the spectrum of pathological retinal changes noninvasively. Using FAF, different patterns of RPE alterations can be distinguished, and this imaging modality can be used to facilitate monitoring disease progression in different phenotypes of CSC [6–13].

For CSC, several patterns of increased and decreased areas of autofluorescence were described in previous studies. The various autofluorescence intensity changes depend on the type of RPE alterations and the chronicity of disease (generally, the more chronic a case is, the more RPE alterations are observed) [12, 13]. Furthermore, in some cCSC cases with residual SRF, a significant discrepancy is observed in the size of the areas of hyperfluorescent abnormalities on FAF versus FA [14]. The aim of the present study was to assess the possible correlation between the findings on FAF and FA in cCSC patients with FAF and FA imaging discrepancies (i.e., difference in size of hyperfluorescent RPE abnormalities on these modalities) at first visit. This study also assessed the predictive ability of FAF for structural hyperfluorescent RPE abnormalities measured by FA over time.

## Methods

### Patient selection

This study was retrospective, non-comparative, and multicentre in design. Data were collected from patients who had presented between January 2003 and December 2015, either at the department of Ophthalmology at Kiel University, Kiel, Germany, or at the department of Ophthalmology at Radboud University Medical Center, Nijmegen, the Netherlands. Approval for this study was obtained both from the Christian Albrecht’s University Institutional Review Board, Kiel, Germany, and the medical ethical committee Leiden-the Hague, Leiden, the Netherlands (since the study was coordinated from Leiden, The Netherlands). All research protocols and data collection were in accordance with the Declaration of Helsinki.

All of the following criteria had to be present in patients to be eligible for inclusion in the study: (1) active course of CSC, defined as presence of SRF on OCT images, presence of “hot spots” of leakage on FA, and corresponding hyperfluorescent areas on ICGA; (2) characteristic findings for chronic course of the disease, with either a history of several CSC episodes, or SRF persistence for more than 6 months; and (3) discrepancy in the size of hyperfluorescent abnormalities on FAF versus hyperfluorescent abnormalities on FA larger than 0.5 optic disc diameters at first visit. Exclusion criteria were any of the following: (1) follow-up of less than 6 months or absence of a control visit; (2) poor quality of the images; (3) presence of either other retinal diseases or conditions that could have caused a decline in visual acuity; and (4) absence of follow-up FA and FAF imaging.

The following information was collected for each patient from the clinical charts: demographic characteristics; documented duration of CSC (time between the moment of diagnosis until the final available follow-up visit); SRF persistence duration (based on available OCT images); number, type, and combination of therapeutic interventions (including photodynamic therapy, retina laser therapy for selectively remodelling of the RPE [15], conventional thermal laser); and best-corrected visual acuity (BCVA). BCVA was measured by using the Snellen visual acuity chart and converted to the LogMAR values for statistical purposes [16]. Data were collected at first and at final visit (after detection of a discrepancy in hyperfluorescent RPE abnormalities of FAF vs. FA).

### Imaging analysis

**Fig. 1:**
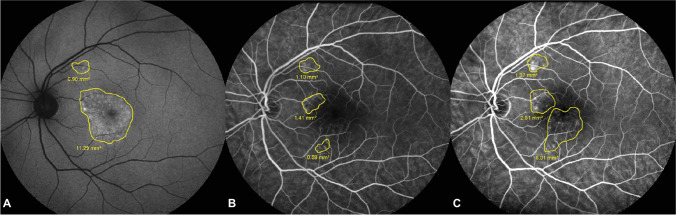
Measurement of corresponding abnormal hyper(auto)fluorescent areas on fundus autofluorescence (FAF) and fluorescein angiography (FA) images using Heidelberg “Draw-Region” tool, in a 40-year-old chronic central serous chorioretinopathy patient, who had a follow-up of 19 months. In this patient, hyperfluorescent retinal pigment epithelium changes are measured on FAF at first visit (**A**), which are larger as compared to hyperfluorescent areas on FA at first visit (**B**) and on FA at last visit (**C**). However, this FA-FAF discrepancy decreased noticeably during the follow-up

All images (OCT, FA, FAF, and ICGA) were acquired by the retinal angiograph HRA2 on Heidelberg machine (Heidelberg Engineering GmbH, Heidelberg, Germany). Measurements of the hyperfluorescent RPE abnormalities were performed in the macular area, defined as the area between the largest temporal arcades. The software “Draw Region” tool in the Heidelberg machine was used to perform the measurements (Fig. 1). The existence of significant discrepancy in the area of hyper(auto)fluorescent abnormalities on FAF and FA images was determined independently by two retinal specialists (V.S., K.P.). A discrepancy larger than 0.5 disc area in the HF-FA between the first and the last visit was considered significant. We measured the area of hyperfluorescent RPE changes as obtained by FAF imaging (which we refer to as: HF-FAF) at the first visit. The final FAF images were also collected and analysed but not included in the current study, due to the relatively frequent occurrence of a mixed (hypo- and hyperautofluorescent abnormalities) pattern, which led to an impossibility to correctly measure changes. Additionally, we measured the area of corresponding hyperfluorescent RPE abnormalities on FA (which we refer to as: HF-FA) at the first and the final visit. The decrease of discrepancy in hyperfluorescent changes between FA and FAF imaging modalities was considered a primary outcome measure.

### Statistical analysis

All data were presented as medians with range (minimum and maximum), as appropriate. The change of discrepancy in size of hyperfluorescent areas was estimated using Wilcoxon rank sum test, and the correlations were evaluated using Spearman correlation coefficients (ρ). Multivariate analyses were performed using a forward stepwise linear regression, where the decrease in discrepancy between the imaging modalities was used as dependent variable and all associated parameters as explanatory variables. The best linear model for grading of discrepancy decrease was calculated. The diagnostic value of each test was assessed by the area under the receiver operating characteristic curve (AUROC). Optimal cut-off values for the two imaging modalities were set at the maximum of total both sensitivity and specificity. Likelihood ratios for the appropriate cut-offs were calculated. A *p* value of < 0.05 was considered to be statistically significant. Data were analysed using SPSS statistics software version 20.0 (SPSS Inc., Chicago, IL, USA) and MedCalc statistical package version 16.2.0 (MedCalc, MariaKerke, Belgium).

## Results

### Patient characteristics

Seventy-one patients (92 eyes) with cCSC could be included in this study. Median age of the patients (58 males, 13 females) at the moment of diagnosis was 42 years (29–72). Median follow-up time was 22 months (6–107), and median CSC duration was 47 months (9–419). Median SRF persistence was 10 months (3–60). Fifty-five percent of the eyes were treated with a monotherapeutic intervention (including photodynamic therapy (29%), conventional laser (3%), selective retina laser therapy (10%), conservative medical therapy (13%)). Median LogMAR BCVA values decreased insignificantly from 0.3 LogMAR (− 0.1–1.3) at first visit to 0.4 LogMAR (− 0.1–1.3) at last visit (*p* > 0.05). Final BCVA correlated significantly with foveal SRF accumulation (*ρ* = 0.724; *p* < 0.001), SRF persistence (*ρ* =  − 0.322; *p* = 0.007), size of HF-FAF at first visit (*ρ* =  − 0.266, *p* = 0.011), and with multiple therapeutic interventions (*ρ* = 0.245; *p* = 0.019).

### Characteristics on multimodal imaging

**Fig. 2:**
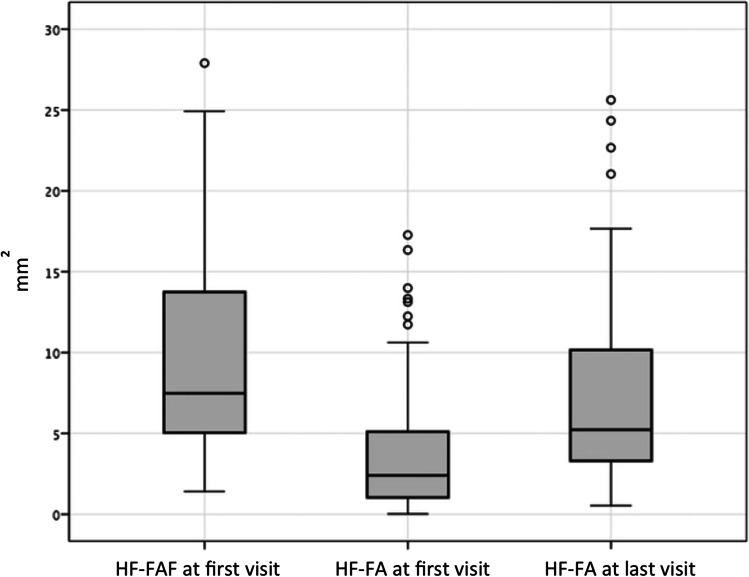
Area of corresponding hyperfluorescent retinal pigment epithelium (RPE) changes on fluorescein angiography (HF-FA) at first and last visit, compared to corresponding hyperautofluorescent RPE changes on fundus autofluorescence (HF-FAF) at first visit. A significant decrease in discrepancy in areas registered by the 2 imaging modalities during the follow-up period is observed (*p* < 0.001)

When comparing the area of HF-FA, the median size increased significantly from 2.40 mm^2^ (0.02–17.27) at the first visit to 5.22 mm^2^ (0.53–25.62) at the final visit (*p* < 0.001). The median area of HF-FAF at first visit was 7.48 mm^2^ (1.41–27.9) (Figs. 1 and 2).

After performing a Spearman correlation test, the following variables correlated significantly with a decrease in discrepancy between HF-FAF and HF-FA: longer follow-up duration (*ρ* = 0.428; *p* < 0.001), larger HF-FAF area at first visit (*ρ* = 0.263, *p* = 0.011), and monotherapeutic approach (*ρ* = 0.245; *p* = 0.019). Among these, longer follow-up duration and larger area of HF-FAF at first visit were the only independent variables associated with a decrease in discrepancy.

**Fig. 3:**
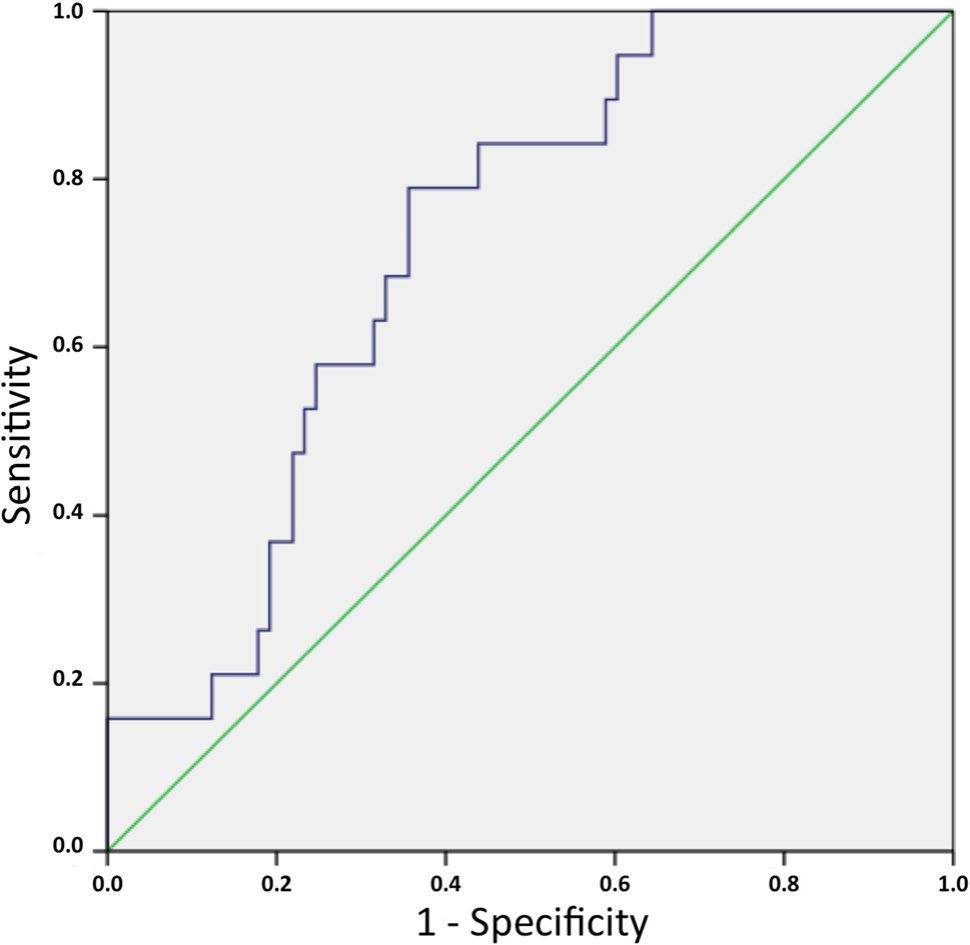
Receiver operating characteristic (ROC) curve, illustrating the diagnostic value of time-dependent fundus autofluorescence/fluorescein angiography discrepancy changes, and comparing the sensitivity and specificity of the model at various threshold settings. The area under the ROC curve (AUROC) = 0.73

A mathematical model in predicting the change of discrepancy between imaging modalities using a forward stepwise linear regression analysis was computed. The best mathematical algorithm for grading RPE abnormalities discrepancy in time was as follows: 0.389 + 0.674 × ((HF-FAF area at first visit) – (HF-FA area at first visit)) – 0.3 × (follow-up duration). All variables in this model were statistically significant with *p* < 0.01 [0.537, 0.527]. In the ROC analysis for this formula, the AUROC was 0.73, which was a statistically significant result, as the confidence interval for AUROC did not include 0.5 (95% CI, 0.614–0.836) (Fig. 3). With a cut-off value of − 2.7, the enlargement of hyperfluorescent RPE abnormalities in FA images could be predicted with 78.9% sensitivity and 64.9% specificity. The proposed model could predict the enlargement of hyperfluorescent RPE abnormalities on FA at final visit in 82.6% of cases, based on HF-FAF abnormalities at first visit.

## Discussion

In the current study, we aimed to find out whether findings on short-wave FAF at baseline could correlate with or predict FA findings in cCSC patients on the long-term. In our retrospective study, we analysed 92 eyes with cCSC with a notable discrepancy in the size of corresponding hyperfluorescent alterations areas on FAF (HF-FAF) and hyperfluorescent alterations on FA (HF-FA) at baseline. We observed that the discrepancy in the size of these areas significantly diminished during the follow-up period, and this decrease independently correlated with the follow-up duration, and the size of HF-FAF at first visit, but not with invasive treatment approaches. Also, no relationship was found with SRF persistence, possibly due to the partial absence of OCT data during the follow-up period in 24% of the cases. Based on current data, we proposed a model which predicted the enlargement of hyperfluorescent RPE abnormalities on FA at final visit in 82.6% of cases, based on HF-FAF abnormalities at first visit.

Different authors previously stated the possibility of using FAF along with other imaging modalities and described correlations between FAF and FA to differentiate between acute CSC and Ccsc [7, 8, 10, 12, 14, 17–19]. It was noted that during the first screening in 72–96% of acute CSC patients, a hypoautofluorescence was registered that corresponded with the leakage site on FA and to the area of serous retinal detachment, which was visualized as a blockage of fluorescein on FA by SRF [9, 12, 13, 20]. At the same time, cCSC patients with persistent SRF showed hyperautofluorescence on FAF imaging that tended to increase in intensity in time (which we refer to as HF-FAF in the current study) [6, 14, 19, 20]. Framme et al. noted that irregular and increased hyperautofluorescence surrounding the original leakage point was registered in about one-fifth of cCSC patients with long-lasting SRF.^18^ These abnormal hyperautofluorescent lesions were also found in the periphery in 57% of (presumably chronic) CSC cases using ultra-widefield FAF, proving the statement that cCSC patients often do not realize when an extramacular serous retinal detachment occurs [20–22].

A possible mechanism has been reported to describe the phenomenon of increased HF-FAF. It was stated that an increased autofluorescence is caused by a higher metabolic activity of the RPE accompanied by accumulation of photoreceptor debris and macrophages, containing fluorophores such as lipofuscin [6, 14, 19]. This abnormal accumulation at the level of the outer segments of photoreceptors and RPE usually precede their degradation and thus can be of predictive value for detection of RPE atrophy in a later disease state, which can occur within the course of CSC [23]. Framme et al. also noted that a clear demarcation of the leakage area based on hyperfluorescent abnormalities was only seen in those patients with more extensive leakage, indicating larger defects of the RPE. These well-defined areas of increased HF-FAF, mostly apparent inferior to the site of RPE changes, were reported in cCSC cases with persistent SRF for more than 6 weeks [24]. We observed the same phenomenon in our study, which underlines the hypothesis that RPE autofluorescence must be enhanced in those areas, because FAF intensity is significantly stronger. An increased HF-FAF in our study was observed even in cases with residual SRF, which should act as a blockade of autofluorescence. HF-FAF in acute CSC generally recovers in 4 to 6 months after the SRF resolution, which is in contrast with findings in cCSC [18].

It was previously stated that hypoautofluorescent abnormalities are associated with worse BCVA and retinal sensitivity, whereas hyperautofluorescent were associated with less RPE damage and, therefore, presumably with a better visual outcome [25], although reduced retinal sensitivity has been reported in cases with hyperautofluorescent RPE changes [17, 25, 26]. In our study, median LogMAR BCVA decreased, although insignificantly, from 0.3 (− 0.1–1.3) at first visit to 0.4 (− 0.1–1.3) at last visit (*p* > 0.05). The RPE alterations on final FAF images in our study could not be interpreted due to the relatively frequent occurrence of a mixed (hypo- and hyperautofluorescent abnormalities) pattern. Therefore, a correlation between FAF abnormalities and BCVA was not analysed. However, this BCVA decline is probably due to the foveal involvement of these RPE abnormalities (Spearman correlation analysis, *ρ* = 0.724, *p* < 0.001).

Based on our findings, we proposed a model that may predict the disease progression in cCSC based on FAF/FA imaging discrepancy. According to this model, larger hyperfluorescent areas on FAF in cases with FAF/FA discrepancy can predict the enlargement of hyperfluorescent areas on FA during follow-up. We may speculate that FAF imaging could especially be used for predictive evaluation of hyperfluorescent RPE alterations in cCSC patients.

The study has several limitations. First of all, it was retrospective in design, and thus we were unable to unveil all influential factors, such as SRF persistence and number of disease recurrences, which presumably could be of importance in the decrease in FAF/FA imaging discrepancy in cCSC. Conducting a retrospective study implicates heterogeneous data, including cases with different characteristics of disease, CSC history, and follow-up duration. We understand that there might have been occurred a selection bias in our study, because we only included patients from tertiary academic referral centres. Therefore, we might have been dealing with more severe cases with complicated disease course and therefore a prolonged follow-up time. Secondly, the measurement of hyperfluorescent abnormalities on FA and FAF images was performed manually, which could have induced a certain error. The threshold of 0.5 disc area that was used to define FA-FAF discrepancy in hyperfluorescent RPE abnormalities is arbitrary. However, this was chosen based on clinical experience and as a reasonable difference that can be detected by ophthalmologists with certain accuracy. Automated or semi-automated software may improve the accuracy of quantitative estimation. An ultra-widefield FA and OCT can be used for monitoring cCSC patients to visualize possible extramacular disease activity. Larger and prospective studies are warranted to prove the predictive ability of FAF in cCSC patients with FAF/FA imaging discrepancy at baseline, and these studies will also contribute to detect further potential factors associated with this possible phenomenon.

In conclusion, at the last follow-up visit in the current study, we found a statistically significant relationship between the areas of hyperfluorescent alterations on FAF and FA in patients with FAF/FA discrepancy at first presentation. Our data show that these changes were time-dependent, which were proved by the proposed mathematical formula.
